# Incidence and risk factors for acute kidney injury after cardiac surgery in a Brazilian University Hospital: a retrospective cross-sectional study

**DOI:** 10.1016/j.bjane.2025.844635

**Published:** 2025-05-01

**Authors:** Pedro Victor Fernandes Ferreira, Heitor J.S. Medeiros, Hugo de Santana Ribeiro Dantas, Eduardo Venancio Teixeira, Lucas Pereira Trevisan, Fernanda de Castro Teixeira, Fernanda Cunha Soares, Raphael Klênio Confessor de Souza, Wallace Andrino da Silva

**Affiliations:** a Universidade Federal do Rio Grande do Norte, Natal, RN, Brazil; b Massachusetts General Hospital, Department of Anesthesia, Critical Care and Pain Medicine, Boston, USA; c Hospital Universitario Onofre Lopes, Departamento de Enfermagem, Natal, RN, Brazil; d Karolinska Institutet, Department of Dental Medicine, Division of Orthodontics and Pediatric Dentistry, Stockholm, Sweden; e Hospital Universitario Onofre Lopes, Departamento de Cirurgia, Disciplina de Anestesiologia, Natal, RN, Brazil

Dear Editor,

Acute Kidney Injury (AKI) is a common and serious complication following cardiac surgery, with reported incidence rates reaching up to 35%. This condition not only increases postoperative morbidity and mortality but also extends hospital stays and elevates healthcare costs. Despite advances in management strategies, the long-term mortality risk associated with AKI remains significant, persisting even a decade after its occurrence. The variability in reported AKI incidence is partly attributed to the absence of a universally accepted definition.^1^

Our study aimed to determine the incidence of AKI among postoperative cardiac surgery patients in a tertiary university hospital in northeastern Brazil. We conducted a retrospective cross-sectional analysis at Onofre Lopes University Hospital, reviewing data from adult patients (aged ≥ 18 years) who underwent cardiac surgery between January 2020 and September 2022. AKI was diagnosed and classified according to the AKIN criteria, based solely on serum creatinine levels. Patients with preoperative serum creatinine levels ≥ 2 mg.dL^-1^ were excluded from the analysis to avoid confusion with pre-existing renal impairment.

To investigate the association between AKI and cardiac surgery, a binary logistic regression model was used. The dependent variable was dichotomized into two categories: patients without AKI and those with a diagnosis of AKI, irrespective of the stage. In the adjusted binary logistic regression model, independent variables with a p-value < 0.20 in the unadjusted analysis were included. A forward selection method was used for model building, starting with a null model and sequentially adding variables until only predictors with a p-value < 0.20 remained. In univariate logistic regression, each independent variable's association with AKI was evaluated individually. Significant associations were identified between all variables and AKI. However, in the multivariate model adjusted for sex and age, only Cardiopulmonary Bypass (CPB) and blood transfusion remained significant.

Among the 277 patients analyzed, postoperative AKI occurred in 91 patients (32.85%). The distribution of AKI stages was as follows: Stage 1 in 19.13%, Stage 2 in 2.53%, and Stage 3 in 11.19% of the cases. The characteristics of patients with AKI after cardiac surgery are presented in Table 1. Several perioperative factors were found to be significantly associated with the development of AKI. Comparative analyses demonstrated that the use of CPB and its duration, the aortic cross-clamping, and blood product transfusions were strongly correlated with an increased incidence of AKI. Patients who developed AKI underwent significantly longer CPB durations (113.9 ± 56.0 minutes) compared to those without AKI (p = 0.011). Furthermore, after adjusting for age and sex, exposure to CPB and blood transfusions was associated with a 2.39-fold and 3.50-fold increase in the risk of developing AKI, respectively (Table 1).

**Table 1:** Characteristics of patients with AKI after cardiac surgery and adjusted logistic regression between characteristics of patients and AKI.

Variables	Categories	n (%) or mean ± SD	p-value
Age (years)		57.93 ± 11.80	0.066^a^
Gender	Male	61 (35.5)	0.236^b^
	Female	30 (28.6)	
Diabetes Mellitus	No	60 (31.6)	0.505^b^
	Yes	31 (35.6)	
Systemic arterial hypertension	No	27 (27.8)	0.192^b^
	Yes	64 (35.6)	
Pulmonary arterial hypertension	No	84 (32.9)	0.914^b^
	Yes	7 (31.8)	
Surgical interventions	CABG	25 (21.9)	0.014^b^
	Valve replacement	39 (37.1)	
	CABG + Valve replacement	5 (55.6)	
	Aortic Surgery	7 (41.2)	
	Other procedures	15 (46.9)	
Blood transfusion	No	31 (19.1)	< 0.001^b^
	Yes	57 (51.4)	
Surgery time (minutes)		240.8 ± 117.5	0.134^a^
CPB	No	24 (19.8)	< 0.001^b^
	Yes	65 (42.5)	
CPB time (minutes)		113.9 ± 56.0	0.011^a^
Aortic cross-clamping	No	30 (22,6)	< 0.001^b^
	Yes	58 (45.0)	
Aortic cross-clamping time (minutes)		90.8 ± 39.1	0.202^a^
		OR (95% CI)^c^	p-value
CPB	No	1	0,010
	Yes	2.,39 (1.23:4.67)	
Blood Products	No	1	< 0,001
	Yes	3.50 (1.88:6.50	

CABG, Coronary Artery Bypass Grafting; CPB, Cardiopulmonary Bypass; SD, Standard Deviation; OR, Odds Ratio; CI, Confidence Interval.

a *t*-test

b Chi-square test

c Adjusted logistic regression (adjusted age and sex).

The AKI incidence rate in our study may be relatively low, considering that we relied solely on serum creatinine levels for diagnosis and stratification. A retrospective cohort study conducted in a similar hospital in Brazil reported AKI incidences of 83.8% and 82.8% using AKIN and KDIGO criteria, respectively, when considering both creatinine and urine output after cardiac surgery.^2^ However, when only serum creatinine was used, the incidence decreased to 27.3% and 24.7%, respectively. Notably, our choice of the AKIN criteria was based on its simplicity and practicality, as it demonstrated comparable diagnostic efficiency to KDIGO in identifying AKI.^3^

There was a strong association between AKI and the type of surgery performed. Combined coronary artery bypass grafting and valve replacement procedures were most strongly associated with AKI development. These combined surgeries are typically more complex and time-consuming. Although no significant association was observed between AKI and surgery duration in our analysis, longer procedures may increase exposure to renal risk factors such as hypoperfusion, inflammation, and prolonged use of devices like CPB, which are commonly utilized in such surgeries.^1^^,^^4^

Surgical factors, particularly CPB and aortic cross-clamping, were significant predictors of AKI. Our findings align with previous studies showing that prolonged CPB and aortic cross-clamping durations increase AKI risk. Blood exposure to the CPB circuit can trigger a systemic inflammatory response, resulting in the release of inflammatory mediators that compromise renal function. Additionally, CPB is associated with renal ischemia-reperfusion injury, where compromised renal blood flow during ischemia and subsequent oxygenated blood reperfusion generates oxygen free radicals, exacerbating renal damage.^4^

Among the intraoperative factors identified, the use of blood products was particularly significant. While blood transfusion can be lifesaving, it carries a substantial risk of renal complications. Previous studies have demonstrated that transfusions may cause hemolysis, increasing levels of nephrotoxic substances such as iron and heme, thereby exacerbating or triggering AKI. Our findings highlight the critical importance of cautious blood transfusion practices during the perioperative period. Strategies to minimize blood product exposure, including evidence-based transfusion protocols and blood management strategies, may reduce AKI risk in cardiac surgery patients.^5^

The study has several limitations, including its retrospective design, the absence of urine output data, and the lack of information on postoperative interventions. The absence of adjustment in the logistic regression for other factors that influence AKI can also be considered a limitation.

In conclusion, our findings highlight the need for targeted perioperative strategies to mitigate AKI risk, particularly through individualized CPB management, restrictive transfusion approaches, and early postoperative renal function monitoring. Although our findings provide valuable insights, a larger cohort and prospective methodologies are needed to enhance the generalizability and robustness of the results.

## Declaration of competing interest

The authors declare no conflicts of interest.

## References

[1] Fuhrman D.Y., Kellum JA. (2017). Epidemiology and pathophysiology of cardiac surgery-associated acute kidney injury. Curr Opin Anaesthesiol.

[2] Silva T.F.D., Silva KRDC, Nepomuceno C.M. (2021). Incidence of acute kidney injury post cardiac surgery: a comparison of the AKIN and KDIGO criteria. Braz J Anesthesiol.

[3] Yaqub S., Hashmi S., Kazmi M.K., Aziz Ali A, Dawood T., Sharif H. (2022). A Comparison of AKIN, KDIGO, and RIFLE Definitions to Diagnose Acute Kidney Injury and Predict the Outcomes after Cardiac Surgery in a South Asian Cohort. Cardiorenal Med.

[4] Kumar A.B., Suneja M., Riou B. (2011). Cardiopulmonary Bypass–associated Acute Kidney Injury. Anesthesiology.

[5] Garg A.X., Badner N., Bagshaw S.M. (2019). Safety of a Restrictive versus Liberal Approach to Red Blood Cell Transfusion on the Outcome of AKI in Patients Undergoing Cardiac Surgery: A Randomized Clinical Trial. J Am Soc Nephrol.

